# Emerging Roles of Propolis: Antioxidant, Cardioprotective, and Antiangiogenic Actions

**DOI:** 10.1155/2013/175135

**Published:** 2013-04-08

**Authors:** Julio Beltrame Daleprane, Dulcinéia Saes Abdalla

**Affiliations:** ^1^Department of Basic and Experimental Nutrition, Institute of Nutrition, State University of Rio de Janeiro, 20559-900 Rio de Janeiro, RJ, Brazil; ^2^Department of Clinical and Toxicology Analysis, Faculty of Pharmaceutical Sciences, University of Sao Paulo, 05508-900 Sao Paulo, SP, Brazil

## Abstract

Propolis has attracted attention in recent years due to its beneficial effects, which make it a potential preventive and therapeutic agent as well as a useful additive in food and cosmetics. The aim of this review is to discuss the growing evidence that propolis may, via a diverse array of biological actions, assist in the prevention of some inflammation-mediated pathologies including cardiovascular disease. The active components of propolis that have been identified so far include polyphenols and flavonoids. These compounds have cardioprotective, vasoprotective, antioxidant, antiatherosclerotic, anti-inflammatory and antiangiogenic actions. Many studies have been undertaken to elucidate the mechanism(s) by which propolis acts, which involve cellular signaling targets and interactions at the genomic level. This review will highlight the effects of propolis that may assist in the prevention of chronic degenerative diseases, such as cardiovascular disease.

## 1. Introduction

The growing market for natural products and alternative medicines has renewed interest in bee products, such as honey, royal jelly, pollen, and propolis [1, 2]. Propolis is the generic name for a complex resinous mixture collected by honey bees from the buds and exudates of various plants. Once collected, this material is enriched with saliva and enzyme-containing secretions and used in the construction, adaptation, and protection of hives [3, 4].

In recent years, many studies of the chemistry of propolis have been published, which reveal that its highly variable composition is influenced by the local flora at the collection site [5–7]. Although many biological activities of propolis are consistently observed, the components responsible vary between geographic and climatic zones [7].

There is considerable evidence on various chemical and biological aspects of propolis, but the therapeutic application and utilization by the pharmaceutical industry are still limited. This is mainly due to the variability of its chemical composition with geographical origin because bees utilize different plants in different ecosystems. Identification of the major compounds in propolis samples is essential; reports of the biological properties of propolis should include a detailed investigation of its composition and botanical sources [7, 8].

The constituents of propolis include polyphenols (flavonoids, phenolic acids, and esters), terpenoids, steroids, and amino acids [9]. There has been extensive research into the composition and biological activities of propolis from various countries [10–13]. Propolis samples from Europe, South America, and Asia have different compositions and therefore different biological activities [12, 14, 15]. However, propolis samples generally show great similarity in their overall composition, regardless of botanical source [15]. Brazilian red propolis has been found in two reports to contain high concentrations of phenolic compounds, 232 mg/g [16] and 257.98 mg/g, respectively [17]. Brazilian propolis also contained higher concentrations of total phenols than samples from other countries: China, 302 ± 4.3 mg/g [18] and 299 ± 0.5 mg/g [19]; Korea, 212.7 ± 7.4 mg/g [20]; Argentina, 187 mg/g [21]; India, 159.10 ± 0.26 mg/g [10]; Portugal, 151 ± 0.01 mg/g [22]; Cyprus, 100.4 ± 7.2 mg/g [23]; and Thailand, 31.2 ± 0.7 mg/g [19]. 

Studies indicate that propolis from Europe and China contains many flavonoids and phenolic acids; tropical propolis generally contains low concentrations of flavonoids [21, 24, 25].

Studies of propolis demonstrate the complexity of its composition and pharmacology; some compounds act independently, while others act synergistically. The therapeutic potential of propolis and its constituents has been the subject of many studies, which have established many pharmacological actions in preclinical testing.

In particular, propolis shows therapeutic potential and may have applications in the pharmaceutical and food processing industries [25–27]. Propolis reportedly has a range of biological activities, including immunomodulatory [28, 29], antibacterial [30], fungicidal [31, 32], anti-inflammatory, healing [33], analgesic/anesthetic [34, 35], and anticarcinogenic effects [36]. 

**Figure 1:**
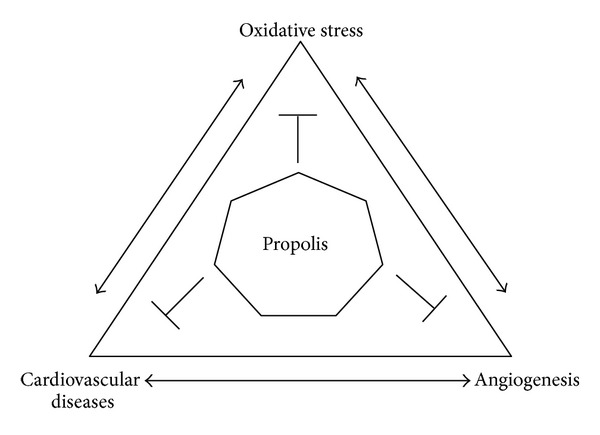
Schematic representation of the relationship between oxidative stress, cardiovascular disease, and angiogenesis, and the effects of propolis on these integrated systems. Propolis influences multiples biochemical signaling pathways, including protective mechanisms reducing events related to chronic inflammatory diseases.

The relationship between oxidative stress, cardiovascular disease, and angiogenesis is well established. Events related to the pathophysiology of angiogenesis and associated cytokines and growth factors can lead to a poor prognosis in many diseases. In fact, chronic cardiovascular disease, oxidative stress, and angiogenesis are strongly associated with one another. In this review, we will present evidence that propolis extracts and their bioactive compounds have antioxidant, cardioprotective, and antiangiogenic activities (Figure 1).

## 2. Antioxidant Activity

It is well established that cellular metabolism generates reactive oxygen species (ROS), such as hydrogen peroxide (H_2_O_2_), the superoxide anion (O_2_^−^), and the highly reactive hydroxyl ion (HO^−^), as well as reactive nitrogen species (RNS), especially nitric oxide (NO). ROS and RNS are ideal signaling molecules because they are locally generated, are highly and rapidly diffusible, and can be neutralized by cellular antioxidants [37, 38]. ROS are usually detoxified by intracellular enzymes, such as glutathione, superoxide dismutase, and catalase [39]. However, unbalanced production and degradation of ROS and RNS can result in accumulation of these reactive species, commonly referred to as oxidative stress. Exposure of macromolecules (lipid, proteins, DNA, etc.) to reactive species results in oxidative modifications with deleterious effects [40, 41].

The antioxidant capacity of propolis may be related to some of its biological effects, including chemoprevention. The flavonoids in propolis are powerful antioxidants, capable of scavenging free radicals and thereby protecting the cell membrane against lipid peroxidation [42]. Moreover, ROS and RNS, together with other factors, are involved in cellular ageing and death in conditions, such as cardiovascular disease, arthritis, cancer, diabetes, Parkinson's disease, and Alzheimer's disease [43–47]. Propolis can reduce cellular levels of H_2_O_2_ and NO, which may be involved in its anti-inflammatory effects [48].

Diverse compounds from propolis have been described as potent inhibitors of oxidative stress. It is well known that the composition of propolis is variable; however, one of its major components, caffeic acid phenethyl ester (CAPE), blocks ROS production in several systems [49]. CAPE has also been identified as one of the major cancer chemopreventive and anti-inflammatory compounds in propolis. *In vitro,* propolis inhibits peroxidation of LDL and nitration of proteins. Moreover, in bovine aortic endothelial cells, propolis was reported to increase eNOS expression and inhibit NADPH oxidase (NOX) [50]. *In vivo,* propolis can increase antioxidant capacity in animals [51] and humans [52], leading to decreased lipid peroxidation, which is strongly associated with the risk of cardiovascular disease [53, 54]. Turkish propolis inhibited hydrogen peroxide (H_2_O_2_-) induced damage to DNA in cultured fibroblasts [55]. The antioxidant activity of phenolic components of the Turkish propolis may reduce damage to DNA induced by H_2_O_2_, which may be related to its chemopreventive activity. Red propolis from Cuba has shown protective effects in models of alcohol-induced liver damage, most likely due to its antioxidant properties [56]. Propolis inhibited macrophage apoptosis via effects on glutathione (GSH) and the tumor necrosis factors/nuclear factor kappa B (TNF/NF-*κ*B) pathway [57, 58]. Moreover, Brazilian propolis from *Baccharis dracunculifolia* modulated 1,2-dimethlyhydrazine (DMH-) induced DNA damage in colon cells [59].

Isla et al. [60] described the protective effect of the Argentinian propolis from different sources against copper-mediated oxidative modification of lipids in unfractionated serum. Five types of Argentinian propolis, collected in different regions, inhibited lipid oxidation during the initiation and propagation phases. All five types of propolis diminished the maximal rate and extent of diene production, indicating that flavonoids can scavenge free radicals, such as superoxide [61, 62], protecting serum lipids from oxidation [60]. Jasprica et al. [52] showed that daily intake of powdered propolis for 15 days decreased the plasma malondialdehyde concentration in men. The extract (0.65 g), available in Croatian community pharmacies, contained 2.5% total flavonoids, equivalent to 16.25 mg of galangin. After 30 days of treatment, an increase in superoxide dismutase activity and changes in red blood cell parameters were detected, including cell count, hemoglobin and mean corpuscular volume, and cell distribution.

The antioxidant effect of Brazilian red propolis has been attributed to chalcones and isoflavonoids (including 7-O-methylvestitol, medicarpin, and 3,4,2′,3′-tetrahydrochalcone) that act as electron donors [63]. Furthermore, total flavonoid content in Brazilian red propolis is correlated with antioxidant activity, suggesting that all the phenolic and flavonoid compounds present contribute to this activity [64]. Chinese red propolis had a higher antioxidant activity than propolis from other sources, which was attributed predominantly to CAPE [65]. Chilean propolis also has antioxidant properties, which are correlated with its chemical composition [66]. Additionally, the antioxidant and free-radical-scavenging properties of propolis may be due to its phenylpropanoid content [67]. Thus, the available data indicate that propolis of different origins and distinct compositions consistently exhibit antioxidant actions. In addition to this antioxidant effect, bioactive compounds in propolis influence a large number of biochemical signaling pathways, and therefore physiological and pathological processes. Antioxidant capacity is one of the most important properties of propolis. Although there are several studies corroborating the potential antioxidant activity of propolis, there is no robust data on the safe dose in humans. Thus, there is need for clinical studies using propolis and its biologically active compounds, including studies of safety and bioavailability.

## 3. Cardioprotective Activity

The modulation of cardiovascular disease markers by propolis has been shown in several studies. *In vitro* and *in vivo* assays have been developed to elucidate the molecular mechanisms of this beneficial effect: regulation of glucose and lipoprotein metabolism; modulation of gene expression; decrease of the activity of scavenger receptors, inflammatory cytokines, and oxidative stress; improvement of endothelial function; and inhibition of platelet aggregation.

Atherosclerosis is a complex process involving the accumulation and modification of plasma lipoproteins in the arterial wall as well as the recruitment and proliferation of immune cells. This process advances through a series of stages beginning with the appearance of a fatty streak lesion, composed largely of foam cells, which are lipid-engulfed macrophages. The fatty streak evolves into a complex atherosclerotic plaque consisting of a lipid core covered by a fibrous cap, with some areas that are rich in inflammatory cells [68–70]. Several authors have postulated that dietary polyphenols reduce the risk of cardiovascular disorders and prevent the development of atheromatous plaques [71–73]. Thus, as a rich source of polyphenols, propolis represents a potential alternative strategy for the prevention of cardiovascular disorders. 

Propolis has been shown to modulate lipid and lipoprotein metabolism. Propolis administration diminished liver cholesterol and triglyceride content and decreased the rate of hepatic triglyceride synthesis in rats [74, 75]. In LDL receptor knockout mice (LDLr^−/−^), treatment with Brazilian red propolis (250 mg/kg/day) decreased levels of triacylglycerol (TAG), total cholesterol (TC), and non-high-density lipoprotein cholesterol (non-HDL-C) [76]. LDLr^−/−^ mice treated with Brazilian green propolis, which is rich in Artepillin C, pinocembrin, kaempferol, or with Chilean brown propolis, which is rich in pinocembrin, CAPE, quercetin, and galangin, also presented low levels of non-HDL-C. Moreover, mice treated with Brazilian red propolis showed significantly reduced TAG and TC, and increased HDL-C, compared to untreated mice. Furthermore, Turkish propolis, which is rich in flavonoids (mainly galangin, quercetin, kaempferol, apigenin, pinobanksin, pinocembrin, and pinostrobin) prevented alcohol-induced acute liver damage and lipid accumulation and induced beneficial changes in the serum lipid profile. HDL levels were high, and LDL levels were low, in mice treated with propolis and alcohol compared to alcohol only [59]. Moreover, propolis also positively affected HDL and LDL levels in rats. Treatment of diabetic rats with propolis of poplar origin diminished levels of total cholesterol, LDL-cholesterol, VLDL-cholesterol, and triglycerides, reinforcing the case that propolis modulates lipid metabolism and may be helpful in syndromes caused by blood lipid abnormalities [75].

In a recent study, the authors hypothesized that propolis may aid in the prevention rather than treatment of atherosclerosis. LDLr^−/−^ mice were treated with distinct polyphenol-rich propolis extracts (250 mg polyphenols/mL/Kg) [76]. Brazilian green, Brazilian red, and Chilean brown propolis reduced the area of atherosclerotic lesions when administered preventively. The strongest inhibitory effect was observed for Brazilian red propolis, which also induced regression of atherosclerotic lesions [76]. Polyphenols from propolis inhibited the progression of atherosclerosis in LDLr^−/−^ mice by improving the lipid profile and by downregulating proinflammatory cytokines, chemokines, and angiogenic factors. Propolis downregulated the mRNA expression of key genes involved in the atherosclerotic process, such as MCP-1, INFg, IL6, CD36, and TGF*β* [76].

It is well known that the modification of the lipid profile is strongly associated with cardiovascular disease [76, 77]. Propolis diminished total cholesterol and elevated HDL-cholesterol in mice. One proposed mechanism of the hypocholesterolemic action of propolis involves the ABCA1 receptor. Many types of propolis upregulate ABCA1 gene expression, which is associated with increased HDL levels; thus, ABCA1 up-regulation may be one mechanism by which propolis improves the lipid profile [76].

An ethanolic extract of Brazilian red propolis (EERP) enhanced ABCA1 promoter activity in THP-1 macrophages [77]. Additionally, cholesterol efflux from macrophages to ApoA-I was significantly increased in a dose-dependent manner by EERP treatment. Thus, EERP significantly enhanced ApoA-I-mediated cholesterol efflux in THP-1 macrophages, which was accompanied by a marked up-regulation of the ABCA1 gene. The effect of EERP on ABCA1-dependent cholesterol efflux may be due to the activation of PPAR*γ* and LXR*α* [77]. In HepG2 and Raw 264.7 cell lines, EEP promoted cholesterol efflux and increased the expression of ABCA1 and ABCG1. Accordingly, C57BL/6 mice treated with 50 mg/kg EEP once a day for 4 weeks by oral gavage showed increased plasma HDL-cholesterol but unchanged LDL-cholesterol [78]. Thus, *in vitro* and *in vivo* data suggest that the beneficial effects of propolis on lipid profile may be one of the mechanisms involved in its atheroprotective effects. This finding suggests that polyphenols from propolis may be useful for the prevention of atherosclerosis.

Platelet aggregation is a major contributor to the atherosclerotic process. Propolis components have shown important effects on platelet aggregation. CAPE (15 and 25 *μ*M) markedly inhibited collagen-stimulated platelet aggregation. As CAPE is involved in various inhibitory pathways influencing platelet aggregation, it may be an important contributor to the potent antiplatelet actions of propolis [79].

NO is an important vasoactive mediator, with vasodilatory and antiaggregative actions that protect blood vessels when released from endothelial cells at low concentrations. However, when NO is produced in high concentrations by inflammatory cells, it may react with other nitrogen and oxygen species, inducing oxidative and/or nitrosative stress. Following the treatment of diabetic mice with poplar propolis, the levels of NO and nitric oxide synthase (NOS) decreased compared to nondiabetic mice [75]. Propolis decreases NO level by decreasing NOS activity, thus protecting blood vessel endothelial cells and reducing neuronal toxicity. Additionally, propolis exerts pharmacological effects by decreasing the actions of NO and PGE2 as well as by reducing the activation of protein kinase in diabetes [75, 80]. Moreover, ethanolic extracts of propolis (EEP) inhibit NO production by decreasing iNOS expression in Raw 264.7 macrophages and by directly inhibiting the catalytic activity of iNOS. The inhibitory effect of EEP on LPS plus IFN-g-induced NO production is mediated either by inhibition of iNOS gene transcription via an action on NF-*κ*B sites in the iNOS promoter or by direct inhibition of the catalytic activity of iNOS [81]. As excess NO production has been implicated in the cardiovascular inflammatory process, the anti-inflammatory activities of EEP may also be mediated by modulation of NO levels.

It is well established that proliferation of vascular smooth muscle cells (VSMCs) is involved in the onset of atherosclerosis. Roos et al. [82] evaluated the antiproliferative activity of CAFE, one of the major components of propolis and honey-derived products, in primary rat aortic VSMCs stimulated by platelet-derived growth factor (PDGF). CAFE inhibited proliferation of VSMCs upon exposure to PDGF in a dose-dependent manner, by interfering with cell cycle progression from the G0/1- to the S-phase. This study indicates that the inhibition of smooth muscle cell proliferation may also be involved in the atheroprotective action of propolis.

## 4. Antiangiogenic Activity

Angiogenesis is the multistep process by which blood vessels are formed. This tightly regulated process involves the migration, proliferation, and differentiation of endothelial cells [83]. Regulation of angiogenesis is absent or aberrant in several diseases characterized by persistent, inappropriate blood vessel development. Inappropriate angiogenesis occurs in more than 80 diseases, particularly in many types of cancer and inflammatory diseases as atherosclerosis [84, 85].

According to Keshavarz et al. [86], green propolis extracts containing artepillin C and CAPE significantly reduced the number of new vessels formed and the expression of metalloproteinases (MMPs) and production of vascular endothelial growth factor (VEGF) from endothelial cells [87]. Different steps of angiogenesis can be affected by propolis and its components. Brazilian propolis and its major component, artepillin C, can inhibit proliferation of human umbilical vein endothelial cells (HUVEC), as well as endothelial cell migration and capillary tube formation, in a dose-dependent manner. Moreover, artepillin C can suppress angiogenesis in both *in vivo* and *in vitro* models, while CAPE inhibits MMP-2, MMP-9, and VEGF activity [87–90].

The effects of Brazilian propolis on HUVEC apoptosis were investigated by Xuan et al. [91]. At a low concentration (12.5 *μ*g/mL), the polyphenols in ethanol extracts of Brazilian propolis decreased the expression of integrin b4 and p53 and the production of ROS. The opposite effects were observed at high polyphenol concentrations (25 and 50 *μ*g/mL), along with depression of mitochondrial membrane potential. Thus, high doses of polyphenols from Brazilian propolis may induce HUVEC apoptosis by acting on the integrin b4 and p53 signaling pathway, resulting in disturbance of mitochondrial membrane potential and increased ROS generation.

Daleprane et al. [92] investigated the actions of polyphenols from Brazilian red propolis on models of angiogenesis. Brazilian red propolis is rich in 1,2,3-trimethoxy-5-(2-propenyl)-benzene, methoxyeugenol, homopterocarpin, medicarpin, 2,4,6-trimethylphenol, 49,7-dimethoxy-29-isoflavonol, 7,49-dihydroxyisoflavone, and 2H-1-benzopyran-7-ol [64]. Brazilian red propolis (10 mg/L) reduced the migration and sprouting of endothelial cells, attenuated the formation of new blood vessels, and decreased the differentiation of embryonic stem cells into CD31-positive cells. Moreover, Brazilian red propolis inhibited hypoxia- or dimethyloxalylglycine-induced mRNA and protein expression of the crucial angiogenesis promoter, vascular endothelial growth factor (VEGF), in a time-dependent manner [92].

Hypoxia is implicated in many inflammatory diseases. The proposal that hypoxia can induce inflammation has gained general acceptance from studies of the hypoxia signaling pathway [93]. Brazilian red propolis decreases accumulation of hypoxia-inducible factor 1 alpha (HIF1*α*) under hypoxic conditions, which in turn attenuates VEGF gene expression [92]. Reduced HIF1*α* protein half-life was associated with increased von Hippel-Lindau (pVHL-) dependent proteasomal degradation of HIF1*α* and reduced Cdc42 protein expression [92].

Brazilian green propolis extract, which is rich in artepillin C, was evaluated by Hattori et al. for its effects on cellular responses to hypoxia [94]. Five compounds that modulated HIF-1 activity were identified. Hydroxycinnamic acid derivatives from Brazilian green propolis inhibited not only HIF-1 transcriptional activity but also hypoxia-induced expression of HIF-1*α* protein and downstream target genes, such as Glucose transporter 1, Hexokinase 2, and vascular endothelial growth factor A. Furthermore, the HIF-1 inhibitors also inhibited angiogenesis. Daleprane et al. [76] investigated the effect of polyphenols from Brazilian propolis on angiogenic gene expression in atherosclerotic lesions of LDLr^−/−^ mice, finding that angiopoietin I, angiopoietin II, VEGF, fibroblast growth factor, metalloproteinases 2 and 9, platelet-derived growth factor, and platelet endothelial cell adhesion molecule were downregulated by polyphenols from Brazilian red and green propolis.

It has been reported that the propolis extracts show antiproliferative activity and that both extracts induced cell death by necrosis [95]. The latter result indicates that certain compounds contained in propolis possess cytocidal activity based on necrosis rather than apoptosis. On the other hand, polyphenols, which are tumor necrosis factor-related, apoptosis inducing ligands, preferentially induce apoptosis in cancer cells and are not toxic to normal cells [96]. These results are therefore not consistent with each other. The inconsistency of propolis activity may be due to the presence of numerous compounds in varying levels, depending on their geographical origin. Generally, biological activity has been assessed by independent groups, making a direct comparison of their work difficult. Propolis-based medicines are often prepared from ethanol extracts of honey hive, as the extracts are generally water insoluble. Further studies are required to establish the quantity and safety control criteria for propolis to allow it to be used safely, and to gain the maximum benefit from its biological activities.

*In vitro* and *in vivo* studies are uncovering antiangiogenic activity in many natural health products, including propolis extracts and their constituents. Further preclinical research is required to determine whether individual compounds or complex mixtures will be optimal for clinical trials. A potential advantage of phytochemicals and other compounds from propolis is that they may act through multiple cellular signaling pathways, acting in different pathophysiological conditions, while also inhibiting angiogenesis and reducing inflammation. Overall, propolis constituents may be helpful as auxiliary therapies for diseases in which angiogenesis must be controlled, such as cancer and cardiovascular disease.

## 5. Perspectives on Propolis Utilization

Propolis contains a broad spectrum of compounds that have many biological activities. It is considered a useful product and is already used in alternative medicine. Recently, there has been a growing interest in its utilization by the food processing, cosmetic, and pharmaceutical industries. Considering this, further studies on the bioactive constituents of propolis are necessary to identify interactions mediating their biological effects. Further studies are also required on their bioavailability, stability in different preparations, and safe and effective doses for prevention or treatment of disease in animals and humans.
